# Humanitarian management of drought needs better water security data

**DOI:** 10.1111/disa.12687

**Published:** 2025-05-13

**Authors:** William A. Veness, Nancy Balfour, Jimmy O'Keeffe, Wouter Buytaert

**Affiliations:** ^1^ Department of Civil and Environmental Engineering and Grantham Institute – Climate Change and the Environment Imperial College London United Kingdom; ^2^ Centre for Humanitarian Change Kenya; ^3^ School of History and Geography Dublin City University Ireland

**Keywords:** anticipatory action, disaster risk reduction, drought, early warning systems, food security, Horn of Africa, water security

## Abstract

Droughts are a primary driver of humanitarian crises in arid regions, yet early warning systems that index humanitarian financing often omit water security data in favour of food security monitoring. Based on 42 expert interviews assessing management barriers and information needs during the 2020–24 drought in the Horn of Africa, resulting in an estimated 71,100 excess deaths in Somalia alone, we find water security data to be critical in shifting management to proactive mechanisms. Monitoring of water availability (such as water quality and groundwater/surface water levels) and water access (such as water prices and household surveys) is needed to design solutions that proactively mitigate water shortages and their secondary impacts on food security (such as through borehole rehabilitation, alternative water supplies, and cash transfers). Furthermore, if causal relations between water and food insecurity are analysed, the cost–benefit basis for financing water supply interventions can be more completely propositioned, and food insecurity hotspots can be better anticipated.

**Practitioner points**

- Local water security data guides proactive water shortage mitigation during droughts.
- Water security data's causal relations enable anticipation of food insecurity hotspots.
- Locally predictive water security evidence provides a more confident cost–benefit basis to finance anticipatory actions.
- This predictive capacity holds significant potential for humanitarian and disaster risk financing applications.

## 1 INTRODUCTION

Underfinanced humanitarian responses to droughts, most notably during Somalia's 2011 drought and famine, prompted calls to shift from reactive to proactive approaches to management (Hillbruner and Moloney, 2012; Sivakumar et al., 2014; Maxwell and Majid, 2016). Various humanitarian early warning systems (EWSs) have been developed to fulfil this need, hosting broad evidence bases of humanitarian indicators that are used to plan and finance humanitarian interventions. EWSs are now the principal information sources for food security monitoring and forecasting in Sub‐Saharan Africa (Maxwell and Majid, 2016; Funk et al., 2019; Andree et al., 2020; Maxwell et al., 2021; Farr et al., 2022). Through these information systems, international organisations, governments, and humanitarian stakeholders trigger finance for management actions described as early or anticipatory when monitored indicators exceed threshold levels (CERF, 2022; Chaves‐Gonzalez et al., 2022; Otkin et al., 2022; Maxwell, Day, and Hailey, 2023).

Following case studies of improved economic and social returns on investment as compared to traditional humanitarian response, EWS‐guided early action has become state‐of‐the‐art for international drought management, with a dedicated anticipatory action funding function now set up by the United Nations (UN)'s Central Emergency Response Fund (CERF) (Cabot Venton et al., 2012; Hallegatte, 2012; Clarke and Vargas Hill, 2013; UNICEF, 2015; UN, 2016; Costella et al., 2017; Cabot Venton, 2018; Guimarães Nobre et al., 2019; Pichon, 2019; Weingärtner and Wilkinson, 2019; Weingärtner, Pforr, and Wilkinson, 2020; UN OCHA, 2021b; Chaves‐Gonzalez et al., 2022). EWSs are evolving rapidly. They are now the subject of an ambitious UN‐led Early Warnings for All (2025) initiative, aiming for universal coverage of multi‐hazard EWSs by the end of 2027. Meanwhile, the recent defunding of the Famine Early Warning Systems Network (FEWS NET) has created critical monitoring gaps in the world's most food‐insecure regions that require the development of alternative systems (Locks et al., 2025).

Prior to these changes in the EWS landscape, the 2020–24 drought and humanitarian crisis in the Horn of Africa exposed the continued, extreme fragility of water, food, and social support systems in this part of the world. Analysis of excess mortality in Somalia between January 2022 and June 2024 produced an estimate of 71,100 (UNICEF, 2025), together with one million excess internal displacements and 23 per cent of the country's population becoming acutely water‐insecure during 2022 (FEWS NET, 2020; UN OCHA, 2021a, 2023a, 2023b; UNHCR, 2021, 2022; IPC, 2022; UN, 2023; WHO, 2023). Arid and semi‐arid lands (ASALs) in Ethiopia, Kenya, and Uganda also reached emergency levels of food insecurity, requiring large‐scale humanitarian disaster responses (IPC, 2022; UN, 2023).

**FIGURE 1:**
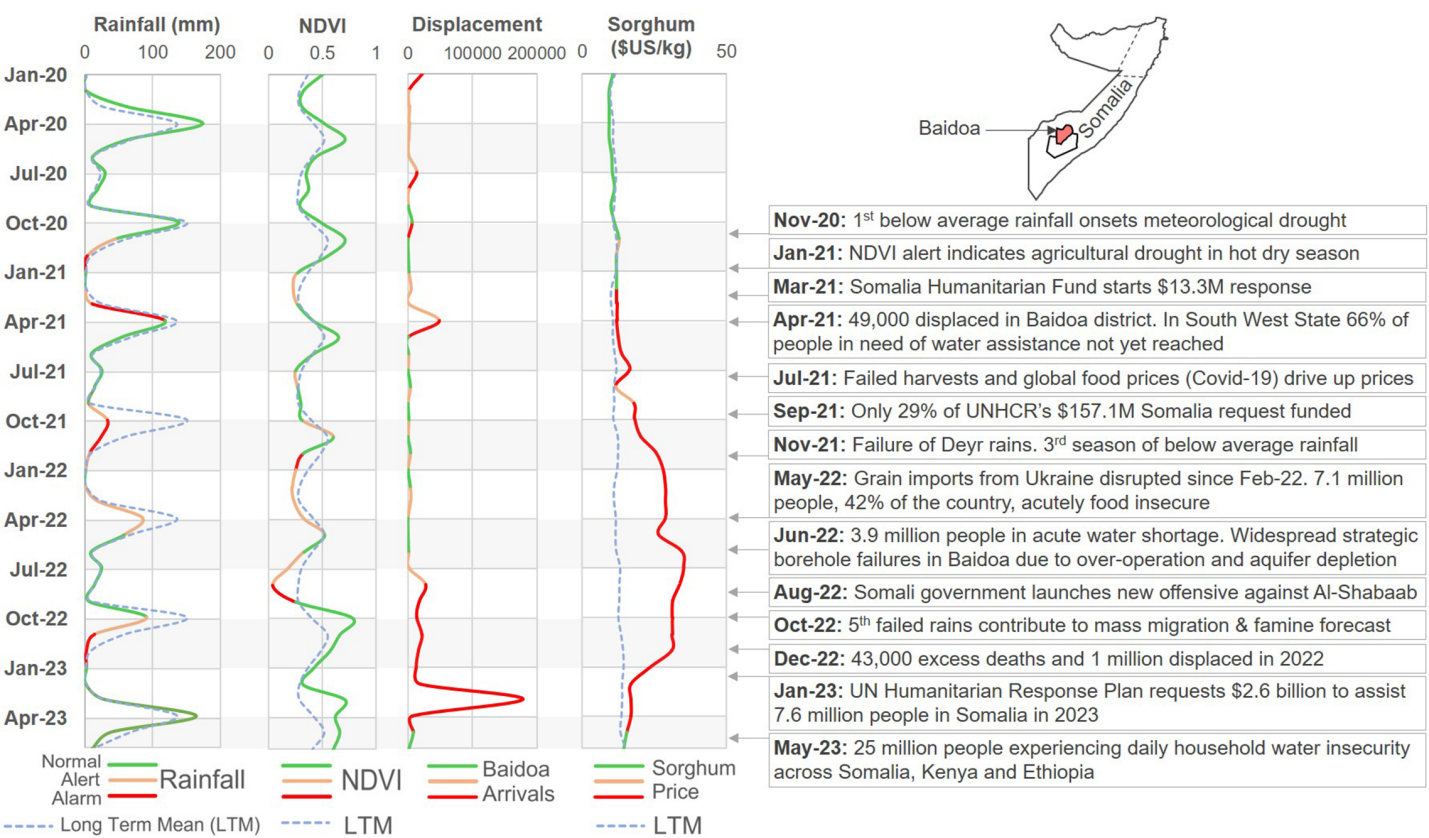
Cascading impacts of drought in Somalia from 2020–23 **Notes:** indicators are plotted from the UN's FSNAU early warning dashboard for Baidoa District (FSNAU, 2025). The colour of each line shows the ‘normal’, ‘alert’, or ‘alarm’ status of that indicator on the dashboard during that month (status criteria are explained in supporting information document S1 in the supplementary materials). A timeline of key events in Baidoa and the rest of Somalia is documented from grey literature (FEWS NET, 2020; UN OCHA, 2021a, 2023a, 2023b; UNHCR, 2021, 2022; IPC, 2022; UN, 2023; WHO, 2023). NDVI = Normalised Difference Vegetation Index; UNHCR = United Nations High Commissioner for Refugees. **Source:** authors, based on FSNAU (2025).

During droughts in the Horn of Africa, water shortages are often first experienced in surface sources and shallow aquifers, which transfer abstraction demand to low‐resilience urban or deep‐aquifer boreholes. Overstressed water sources then have an increased risk of mechanical failure, low yields, or complete water depletion (Kebede et al., 2017; Foster et al., 2020; MacAllister et al., 2022). Subsequent water shortages precipitate a range of severe health and lifestyle impacts (REACH, 2022), as well as cascading secondary impacts on food security. They directly cause food shortages through the loss of livestock and reduced crop yields (Farr et al., 2022; Warsame et al., 2022), while displacing populations towards migration centres when water points fail (Lindley, 2014). Growing demands on remaining infrastructure and resources then increase water and food prices, disease, and civil insecurity risk, which further pressurise local water and food security in a positive feedback process (Wilhite, Svoboda, and Hayes, 2007; Postel, 2017; Funk, 2020; Lin, Qian, and Schubert, 2022). These sequential impacts are apparent in monitoring data from the early warning dashboard of the UN's Food Security Nutrition and Analysis Unit (FSNAU) during the 2020–24 drought: rainfall (water availability) deficits in Baidoa District, Somalia, preceded increases in vegetation stress, internal displacements, and market prices (see Figure 1). These effects, as in the 2011 Horn of Africa drought (Little, 2012; Maxwell and Fitzpatrick, 2012), were compounded by other factors, including global food price rises, political elections, and domestic conflict (Gumucio, Greatrex, and Lentz, 2022; Majid et al., 2022).^1^

**FIGURE 2:**
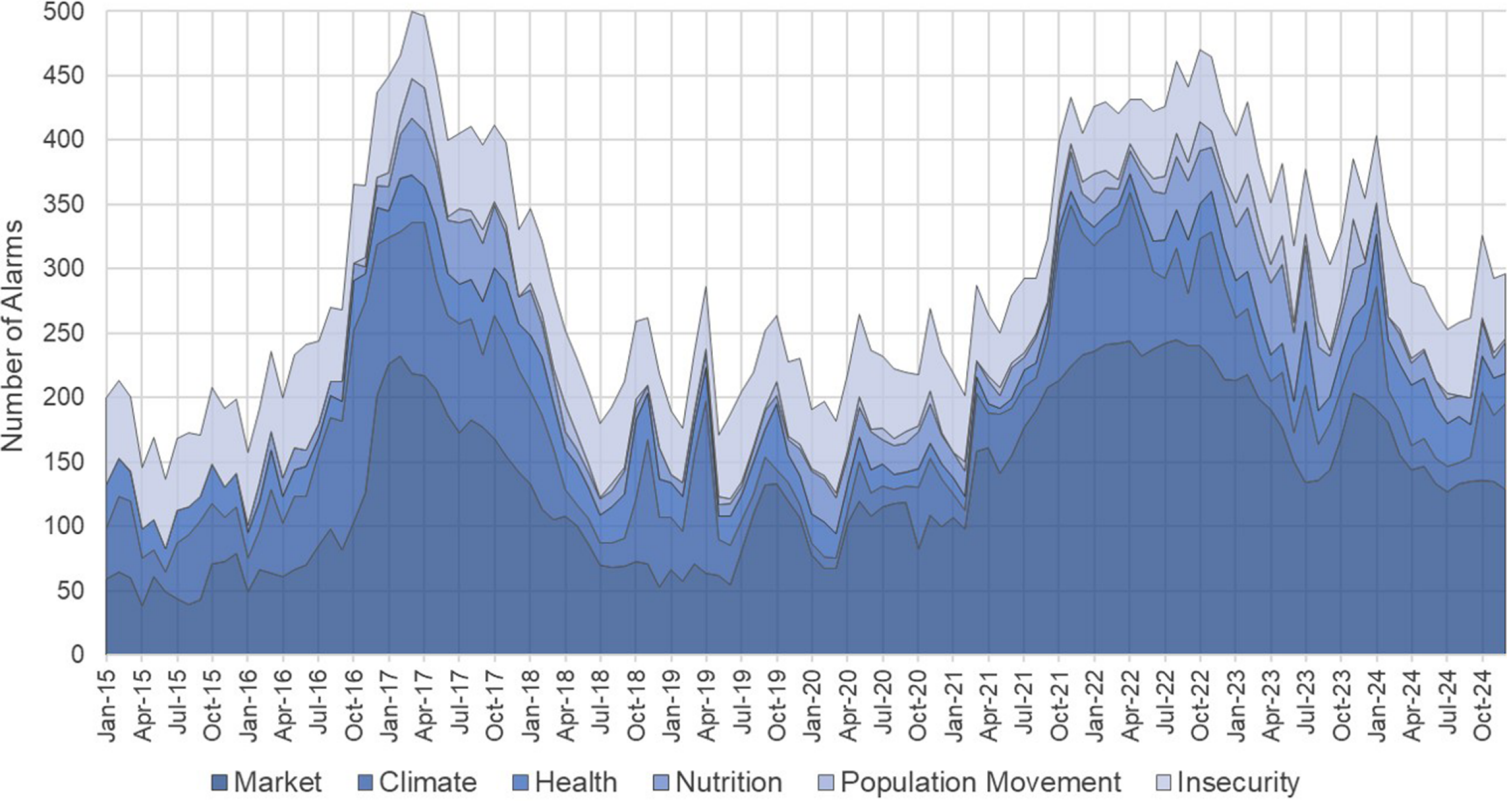
The number of food security indicators above FSNAU's ‘alarm’ threshold across Somalia's 74 administrative districts, 2015–24 **Source:** authors, based on FSNAU (2025).

Anticipatory actions did not receive significant financing relative to the scale of humanitarian response during the early stages of drought (Chaves‐Gonzalez et al., 2022; Farr et al., 2022; Majid et al., 2022). Funding for water, sanitation, and hygiene (WASH) assistance remained especially low, with such financing requests less than 40 per cent fulfilled throughout 2021–22 (Ikeda, 2021; UN OCHA, 2023b). Subsequently, the financial resources committed to food security exceeded WASH assistance 10‐fold in Somalia's UN‐coordinated *Humanitarian Response Plan* (Kellett and Caravani, 2013; Ikeda, 2021; Mourad, 2023). Solutions for water insecurity are underfunded despite evidence that it increases food insecurity risk through, for example, population displacement, migration, crop failure in irrigated systems, and loss of livestock (Lindley, 2014; Anderson et al., 2021; Young, 2021; Farr et al., 2022; Krishnamurthy et al., 2022; Warsame et al., 2022). The strong relationship between drought (anomalous deficits of water) and food insecurity is evident in longer‐term FSNAU data (see Figure 2): the number of humanitarian indicators in the ‘alarm’ phase across Somalia during the documented 2016–17 and 2020–24 droughts doubled as compared to non‐drought levels (‘alarm’ criteria are explained in supporting information document S1). A narrower set of indices monitored during the 2011 drought showed equally acute increases in market, nutrition, insecurity, health, and population movement indicators of food insecurity relative to non‐drought levels (FSNAU, 2011).

The scale of drought's contribution to food insecurity in the Horn of Africa has led to calls for a disaggregation of the management of water shortages and their effects from regular food security management processes (Calow et al., 2010; Young, 2021; Action Against Hunger, 2023). A more explicit mitigation of water insecurity through water resources management (WRM) interventions can mitigate the cascading secondary impacts of water shortages, with high positive returns on investment (Calow et al., 2010; Guimarães Nobre et al., 2019; Weingärtner and Wilkinson, 2019; Weingärtner, Pforr, and Wilkinson, 2020; Young, 2021; Chaves‐Gonzalez et al., 2022; Action Against Hunger, 2023; Warsame, Frison, and Checchi, 2023).

### 1.1 The issue of water security data scarcity

Water security monitoring has not received the same commitment as food security monitoring in low‐ and middle‐income countries (Farr et al., 2022; Majid et al., 2022; FSNAU, 2025). Its common definition mirrors the concept of food security, as being the product of the same four domains: availability; access; use; and stability (Cook and Bakker, 2012; Slaymaker et al., 2020; Young, 2021). Availability refers to the physical presence of water in reserves; accessibility is the ability to retrieve the water for consumption; use describes the safety of the water for its desired applications; and stability is the continuity of the three preceding domains (Slaymaker et al., 2020; Young, 2021). Information on water resource availability in the Horn of Africa is often limited to low‐resolution, satellite‐based measurements of rainfall, soil moisture, reservoir area, and vegetation health, owing to a scarcity of in‐situ hydrological measurements such as groundwater levels, river levels, or local rainfall (Wilhite, Sivakumar, and Wood, 2000; Gure, 2021; Mardian, 2022; Veness and Buytaert, 2025). Socioeconomic indicators of water access and water prices or indices from household surveys such as the Household Water Insecurity Experiences Scale (HWISE–RCN, 2025), are not routinely collected by EWSs or other humanitarian information platforms (Bedrosian et al., 2021; Young, 2021; Zaki, 2021; REACH, 2022). The scarcity of water monitoring data is a recognised barrier to WRM (Garrick et al., 2017; UNESCO World Water Assessment Programme, 2021; Veness et al., 2022), but its implications for disaster risk management of drought are still unclear.

**FIGURE 3:**
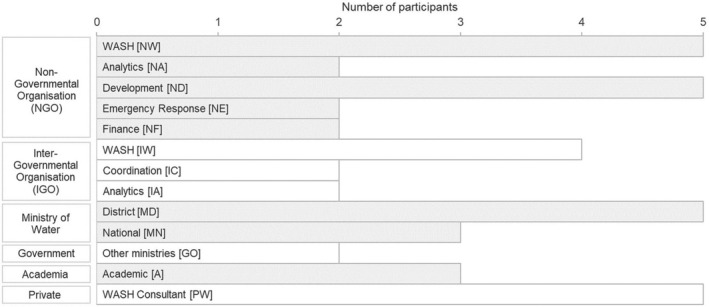
Professional roles of the 42 study participants **Notes:** the profession reference code used to anonymise interviewees in the analysis is shown for each role. Full stakeholder group descriptions are included in supporting information document S2. **Source:** authors.

To understand better the impact of information scarcity in drought management, we interviewed 42 expert regional stakeholders during the 2020–24 drought in the Horn of Africa, with participants ranging in decision‐making level from district water engineers to government and humanitarian financing stakeholders (see Figure 3). This sample captured perspectives from a representative range of the key disciplines and sectors involved in the management of drought; a deliberate effort was made to strike a balance between those with WASH experience (22 participants) and those without it (20 participants)—full descriptions of the stakeholder groups interviewed are available in supporting information document S2. The interviews were analysed using interpretive thematic analysis, which served to generate the thematic plots, qualitative analysis, and conceptual diagrams presented in this paper.

Our results first present the expert‐perceived barriers to drought management during the Horn of Africa crisis to situate better the issue of water monitoring data scarcity and its relations with other barriers. Next, we assess stakeholder information needs for addressing barriers and completing respective drought management activities and objectives, identifying various information needs pertaining to water security data among both WRM and financing stakeholders. We conclude by discussing the potential role of local water security data in tackling the financing barriers to drought‐related disaster risk management.

## 2 METHODS

A semi‐structured interview topic guide was used to collect qualitative perspectives from participants (Galletta, 2013; Patton, 2015; Saunders et al., 2015; O'Keeffe et al., 2016; Brinkmann and Kvale, 2018; Gumucio et al., 2021; Gumucio, Greatrex, and Lentz, 2022). Given the specialist expertise and diverse perspectives of the study participants (see Figure 3), this was an appropriate way to pursue emergent information through multiple follow‐up questions (Galletta, 2013; Mojtahed et al., 2014). The two‐way, discursive nature of the interviews ensured a style that emulated regular conversation, and it supported the co‐construction of findings between interviewer and participant based on their subjective experiences (Mills, Bonner, and Francis, 2006; Creswell, 2009; Mojtahed et al., 2014; Patton, 2015).

Prior to the interview's design, literature reviews and unstructured discussions with drought stakeholders were conducted to establish objectives, methods, and potential questions (Saldaña, 2021; Veness, 2024). These prior stages identified drought management and information systems as a research area for interrogation, given a scarcity of academic literature evaluating their design and prior stakeholder discussions, raising concerns about their existing role in the drought management system (Maxwell et al., 2021). Somalia and the rest of the Horn of Africa were chosen as the key focal points for discussion and participant sampling, given their recent history of drought (in 2011 and 2017). The interviews focused specifically on the ASALs within Somalia, the self‐declared independent republics of Puntland and Somaliland, and eastern Ethiopia; Kenya and northeastern Uganda were also spotlighted, as the cross‐border‐connected region shares similarities in climates and livelihoods (Hughes, 2019; Bjornlund, Bjornlund, and van Rooyen, 2020; Thomas et al., 2020). There are limitations to generalising some of the analysis across the region, such as an under‐representation of frequent conflict zones, political differences, local disparities in livelihood vulnerabilities, and areas of more acute water scarcity (Krampe et al., 2020); however, the similarities are appropriate for collective analysis that required a regional focus to capture the perspectives of important humanitarian stakeholders that professionally operate at the regional level, such as non‐governmental organisation (NGO) finance, humanitarian, and analytics stakeholders. The 2020–24 drought, which was experienced in the entire study region, provided a unique opportunity to interview stakeholders during its progression (in 2022), with all of them drawing upon the ongoing event (UN OCHA, 2021a; UNHCR, 2021). The depth of the insights and the accuracy of the information shared benefitted from participants being able to recall recent experiences in detail (Tuckey and Brewer, 2003).

An open, introductory question (2) inductively assessed participant‐perceived barriers to effective drought management, helping to frame information system‐related barriers within the wider barrier network in the later analysis. Ensuing questions then centred on monitoring information systems and information needs for the management of droughts:

1. What is your professional role and experience relevant to droughts?
2. What are the barriers to better management of droughts?
3. What information do you use for early warning and monitoring of droughts?
4. What evidence do you need to take drought management actions? What early actions do you and/or your institution take?
5. What evidence do you need to release funding of drought‐associated actions at your institution? (If relevant.)
6. Do existing information sources meet your/your colleagues' information needs?
7. What further information would improve your/others' actions taken? How?

Snowball sampling began following ethical clearance by Imperial College London, United Kingdom; key informants were identified from contacts provided by project partners: Concern Worldwide, the UK's Foreign, Commonwealth & Development Office (FCDO), the Norwegian Refugee Council, the Building Resilient Communities in Somalia (BRCiS) consortium, the Ministry of Water Resources Development in Somaliland, and the Food and Agricultural Organization of the United Nations' Somalia Water and Land Information Management (SWALIM) unit. All participants had at least five years of professional or academic experience in the Horn of Africa, with more than one‐half of interviewees having at least 20 years' experience. All of them were actively studying or managing the Horn of Africa drought in 2022, although many experts from academia, the private sector, NGOs, and the UN also drew upon global experiences. Snowball sampling purposefully targeted experts from diverse backgrounds; efforts were made to include a range of professions to capture a participant population representative of the main expert stakeholder groups in WRM, drought governance, and the humanitarian sector in East Africa (Gumucio et al., 2021). None of the participants were previously known to the authors. Interview sampling was discontinued once the amount of new information arising from answers became low (Galletta, 2013; Saldaña, 2021).

**FIGURE 4:**
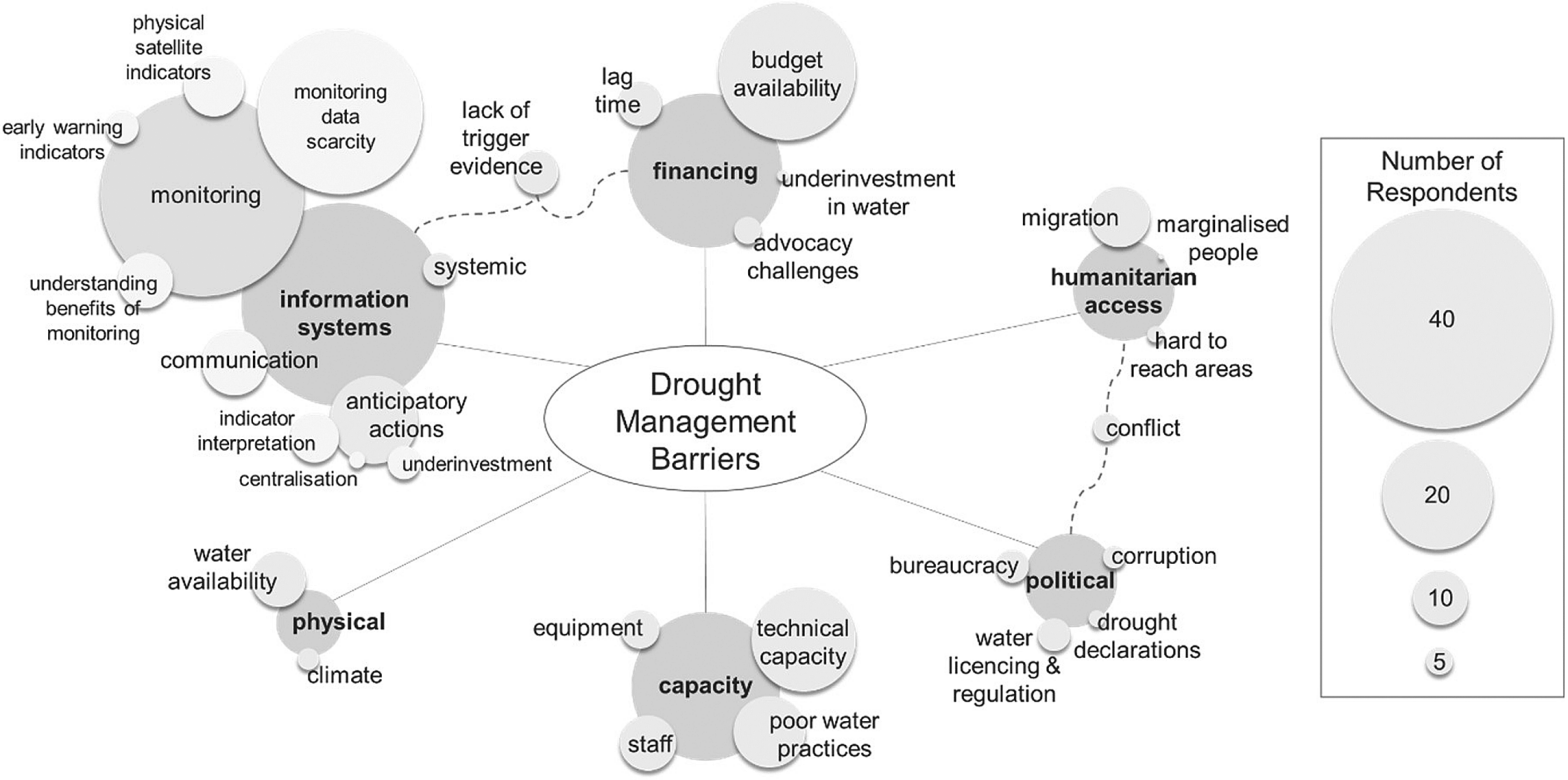
Barriers to managing drought impacts as perceived by experts during the 2020–24 drought in the Horn of Africa **Notes:** themes and sub‐themes from the thematic coding analysis are shown in the figure, scaled as per the number of respondents coded as in that category. An extended discussion of the barrier themes is provided in supporting information document S3. **Source:** authors.

**FIGURE 5:**
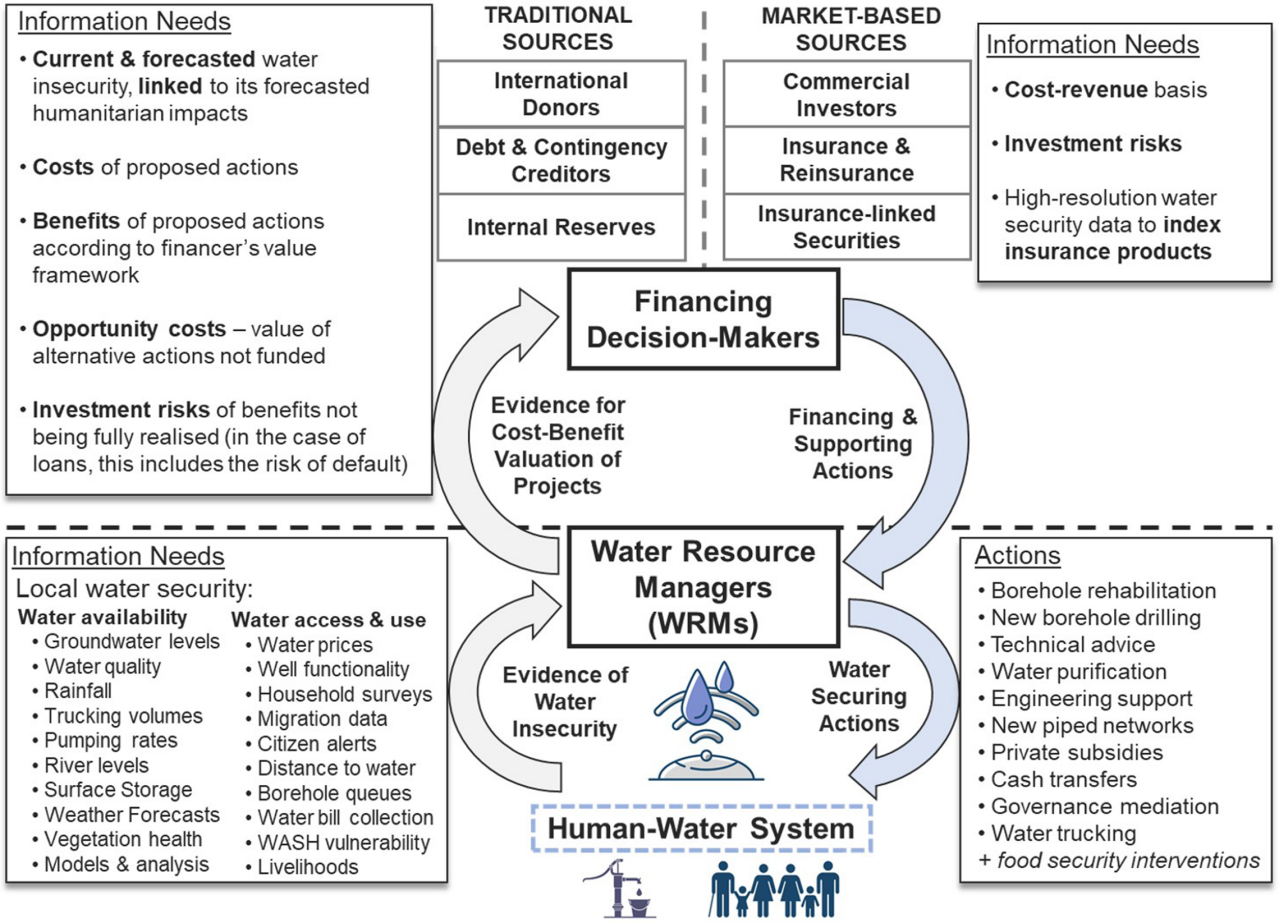
An interpretive conceptual framework for water security‐centred decision‐making in disaster risk management of drought **Notes:** in the framework, information needs are met for the rapid approval of proactive, evidence‐based actions focused on water security. Water resource managers are the primary users of water security data for planning direct actions that manage water resources and supply systems. Financial decision‐makers from traditional sources require evidence to justify financing decisions on cost–benefit bases, and they use a decision‐making frame that considers broader development and humanitarian objectives as well as competing project proposals. Market‐based finance sources include index‐based sources, which trigger financial payouts once selected indices exceed predetermined thresholds, as well as commercial investors in water infrastructure who seek profitable returns at low risk. The listed information needs and actions represent codes recorded during the thematic analysis. **Source:** authors.

**FIGURE 6:**
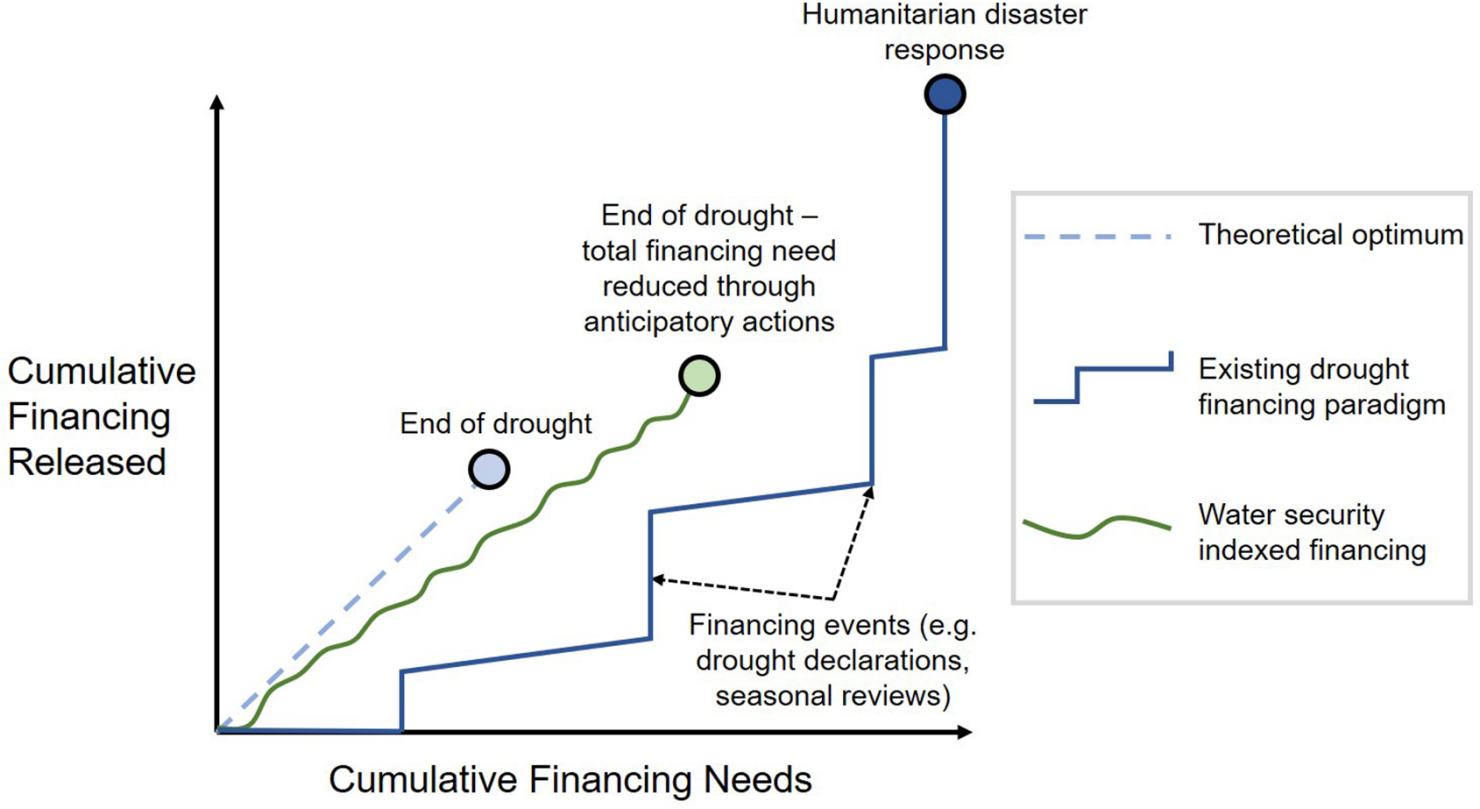
Conceptual model of financing efficiency, comparing drought financing indexed by high‐resolution water security data with traditional approaches to drought financing guided by humanitarian EWSs **Source:** authors.

Interviews were conducted on a one‐to‐one basis via video conferencing software (36) or in‐person in Hargeisa, Somaliland (3), Nairobi, Kenya (2), and Kampala, Uganda (1). Video conferencing was preferred in many cases to enhance participant access and availability, and, as participant responses can sometimes be less detailed online, a one‐hour timeslot was reserved for follow‐up questions (Johnson, Scheitle, and Ecklund, 2019; Lobe, Morgan, and Hoffman, 2022). In all instances, the interviews were conducted in private spaces using the same pre‐interview information, consent, and data management procedures and following the ethical standards of Imperial College London. All interviews were recorded, transcribed, and anonymised before thematic qualitative coding analysis, which used an inductive coding approach for all questions (Patton, 2015; Saldana, 2021). Interpretive codes were assigned to key question‐relevant information within the transcriptions and organised into emergent themes of related codes using NVivo software. Three rounds of coding were completed to standardise codes, themes, and sub‐themes across the study (Saldaña, 2021), which have been qualitatively analysed in the results. A thematic plot (see Figure 4) has been designed to quantify the number of participants referencing codes, themes, and sub‐themes in relation to Question 2, and conceptual diagrams (see Figures 5 and 6) have been constructed for the discussion to communicate principal interpretations from the thematic coding analysis (Mills, Bonner, and Francis, 2006; Patton, 2015).

## 3 RESULTS

### 3.1 Drought management barriers

Thematic analysis of expert‐perceived barriers to drought management organises them into six themes (see Figure 4). Information systems are the modally referenced theme, within which experts emphasise the impacts of water monitoring data scarcity on management among both WRM stakeholders (see subsection 3.1.1) and humanitarian financing stakeholders (see subsection 3.1.2). Our respondents also discussed the barriers of financing, institutional capacities, and politics and humanitarian access issues related to domestic conflicts. While we focus on information systems in this article, a full analysis of the six themes and their relative roles as barriers is available in supporting information document S3.

#### 3.1.1 Information system barriers for WRM stakeholders

For stakeholders in WRM, including governing water authorities, NGOs, community committees, water utilities, and private water suppliers, the absence of local hydrological monitoring data collection inhibits their capabilities to anticipate and mitigate water shortages (A3, MD3, MD4, MN1, ND4, NW4, PW5, IC1). As one study participant (IW1) remarked:

*We want to be telling people what is happening from the data and say, ‘hey, in this region, we have these 10 boreholes that are not working, or in that region that is where they are most affected’. There's only one borehole that is functional, it has this much water, it's pumping for this long and we need to fix it before X, Y, and Z happens*.

For example, many WRM respondents believe that a scarcity of monitoring of groundwater level and quality prevents water shortages from being routinely anticipated by authorities or operators before borehole supply failures occur (MD3, ND2, ND3, NF1, NW1, NW3, PW5, IA1, IC2) (Veness and Buytaert, 2025). This was evident in Baidoa District, Somalia, in 2022, where collective negligence of water monitoring and over‐pumping caused flash increases in water insecurity following aquifer depletion at key refugee sites (PW1, IW2, NW1) (Giri, 2022; SWALIM, 2022; Balfour, 2023). An absence of groundwater monitoring data also makes the long‐term identification of productive aquifer sites for new borehole construction challenging, which compromises their quality and reliability (MD2, MD3, MD4, MN3, ND2, NF2, NW2, NW5, IW2). Six of our respondents (NW1, IA1, GO1, MN3, MD2, A1) from a range of backgrounds in WASH (3), government (1), academia (1), and analytics (1) all emphasised that supply failures in poorly‐designed water infrastructure are at the core of cascading impacts in early drought phases, generating immediate water insecurity, displacement, and secondary pressures on food security (such as acute food insecurity among the displaced, the creation of demand hotspots at migration sinks, abandoned crop harvests, and the sale of livestock) that substantially increase humanitarian management needs. One of them (NW1) stated that:

*It is cheaper to assist people who are lacking water [in‐situ] than to wait until they migrate. Otherwise, their needs are going to increase; maybe they now need shelter, they will need food, they need other things. So, we want to try and actually be proactive in that way*.

#### 3.1.2 Information system barriers for humanitarian financing stakeholders

Our respondents underlined associations between information system and financing barriers. As humanitarian financing is largely triggered by EWSs that prioritise food security data, the funding of drought‐associated projects only commences at scale once water shortages have augmented socioeconomic stress to a level detectable by food security survey instruments (PW1, A1, NA1, NA2). One expert (A1) described the humanitarian system as being ‘enamoured with food security’, and seven stakeholders in high‐level humanitarian management or advisory roles (A1, MN3, NA1, NA2, ND1, IA1, IC2) noted an unwillingness to fund significant drought‐associated actions until real‐time food security turns red on district‐scale maps. Another participant (PW1) said:

*A problem with the current system is that it's too broad brush. You know, the indicators will give you an error on a district‐wide approach, but it won't tell you that this particular community is going to go downhill first or, you know, this is what's going to happen here*. … *I think that much more localised monitoring, with local triggering of local action, is what's really needed*.

Without local water monitoring, there is often insufficient evidence to release financing for drought‐associated projects ex‐ante (IC2, NF1). Lacking such data, many respondents (IC2, NF1, NF2, A1, A2, NA1, NA2, ND2, ND3, NE1, NE2, NW1, PW1) stressed that it is difficult to forecast confidently how drought and water insecurity will affect local humanitarian needs and, subsequently, the expected value of mitigation projects (Wilhite, Svoboda, and Hayes, 2007). Devoid of confident project value propositions to attract early finance from limited capital pools (Basolo et al., 2009), donor and creditor finance is instead channelled to other regions, countries, or humanitarian crises where there is clearer evidence of active need and potential project returns on an investment (A1, NE2, NF2, IC2, IA1). In the words of two participants (NA1 and IC2, respectively):

*The humanitarian sector in general doesn't like the idea of uncertainty. They need something to be concrete, yet early warning early action is, inherently, embracing uncertainty in some ways*.

*Donors do not give us give us a lot of funding, because, [with] anticipatory action, it's very difficult to prove the result. It's not really visible without the right data*.

Market‐based alternatives for contingency financing, particularly parametric insurance and catastrophe bonds, have also been constrained by the scarcity of water monitoring data because their economic viability requires accurate and confident indices to trigger payouts at sufficiently low risks of impact miscalculation (NF1) (Clarke and Vargas Hill, 2013; Durand et al., 2016; Okpara et al., 2017; Ikeda, 2021). The existing lag time between physical drought onset and action financing is widely perceived to create a window of unmitigated water shortages and secondary impacts, including water insecurity, food shortages (A1, NA1, ND2, ND3, ND5, IA1, NF1), and displacements (IA1, PW1, NW1, A1, MN3, ND3, NF1, NW2, PW2), which all compound to increase a drought's overall management costs and humanitarian needs (IW3, NE1, PW3, PW4, PW5, IA1, NA1).

### 3.2 Drought management information needs

Many respondents described information needs for a range of improvements in the resolution, accuracy, analysis, and communication of the existing socioeconomic and satellite‐based monitoring indices available on EWSs and other humanitarian networks; however, 76 per cent of them further identified unmet needs for local data on water security. Our thematic analysis finds that water security information has a different function for WRM practitioners and the decision‐makers who finance WRM, development, and humanitarian activities.

For WRM practitioners, the benefits of high‐resolution water security data are largely the inverse of their perceived information system‐related barriers, that is, real‐time water security data can enable real‐time anticipation of water access issues, while also enabling more sustainable, evidence‐based, long‐term development of water supply systems. For financing decision‐makers, whom we analyse in this section, our respondents pinpointed the potential for water security data to improve the timing, efficiency, and scale of investments to manage drought risk. In doing so, they differentiated between traditional and market‐based sources of finance. Traditional sources (see subsection 3.2.1), such as development banks, government reserves, NGOs, and international donor funds, often consider social benefits alongside economic returns, and they are generally competitive and constrained sources of finance (MN1, IC1, IC2, IW3, NF1) (Ikeda, 2021). Market‐based sources (see subsection 3.2.2), such as insurers, reinsurers, commercial banks, and private corporations, principally seek a positive economic return on an investment and are viewed as a promising solution to existing shortages in humanitarian, WRM, disaster risk reduction, water utility, and development financing (NF1, IA1, ND2) (Clarke and Vargas Hill, 2013; Durand et al., 2016; Timmons Roberts et al., 2017; Fava and Vrieling, 2021).

#### 3.2.1 Traditional sources of finance

In traditional pathways of humanitarian and WRM financing, project finance is reviewed using cost–benefit analysis (CBA) or similar methods, requiring that the forecasted benefits of finance exceed the costs, while presenting a sufficiently low risk of project failure (IC2, NF1, NF2, A1, A2, NA1, NA2, ND2, ND3, NE1, NE2, NW1, PW1) (Pandeya et al., 2016; Dasgupta, 2021). Furthermore, as the demand for finance generally outstrips supply, the value proposition of a project (according to the unique objectives and criteria of the financing institution) should also exceed that of alternative requests competing for financing (IA1, IA2, NF1, NF2, IC1). Unfunded requests are therefore opportunity costs for the funded project (IA1). As one participant (IA1) put it:

*For the most part, their decision is mostly on more money, less money, right? Do we give more money to Somalia, and less to South Sudan, or whatever. So, [humanitarian stakeholders] gear their huge early warning systems to be able to, you know, decide on that specific lever*.

The format of this CBA process varies in formality, degree of quantification, and nomenclature between organisations. Within international NGOs, project CBAs can be rigidly quantified and formally reviewed, whereas governmental and intergovernmental committees may deliberate allocations more discursively, guided by CBA evidence, but allowing for greater consideration of personal, political, and holistic decision‐making elements that are less easily quantified (IA1, IA2, NF1, IC1, A1). One participant (NF1) stated:

*In the East Africa region, a lot of that [FSNAU and FEWS NET] data was interpreted by the FSNWG [Food Security and Nutrition Working Group], and a lot of that data has been used to inform CERF's decisions to release funds in the East African region*.

Thirteen respondents noted a demand for more local water security data specifically to improve the evidence base available for value propositioning projects before and during droughts (IC2, NF1, NF2, A1, A2, NA1, NA2, ND2, ND3, NE1, NE2, NW1, PW1). The data can be used to assess real‐time trends in water security and infrastructure, forecast the expected humanitarian impacts of any changes, and subsequently propose the expected benefits of water‐securing actions that mitigate those impacts (IC2, ND5, PW4) (Basolo et al., 2009). In this process, analysing local correlations and causal relationships between water insecurity and other humanitarian needs is critical to propositioning the full potential benefits of WRM projects (A2, MN2, NA1, NE2, NF1, PW5, IA2, IC1, IC2, IW1) (Wilhite, Svoboda, and Hayes, 2007; Maystadt and Ecker, 2014; Pandeya et al., 2016; Ringler et al., 2018; Pape and Wollburg, 2019; Ngcamu and Chari, 2020; Dasgupta, 2021). One participant (NF1) remarked:

*Historical analysis is also going to be good for forecasting. If the groundwater level is below [the] historical average, and you know the [humanitarian] impacts were bad at that certain level in the past, then that information becomes useful for us*.

Five respondents with humanitarian analytics or financing backgrounds (NF1, NF2, IA1, IA2, NA1) stated that these relations can enable water security to become a more reliable predictor of food insecurity and other humanitarian needs, to index routinely the finance of anticipatory action projects with a focus broader than WRM (Anderson et al., 2021). Some anticipatory action projects already use hydrological data as predictive triggers, but their dependence on lower‐resolution, satellite‐based indices of rainfall, soil moisture, and vegetation creates a high index uncertainty that prohibits their upscaling (A1, ND2, ND3, ND5, NE2, NW4, PW3, IA2, IC1, IW1, IW2, NF1) (Brown, 2013; Wilkinson et al., 2018; Guimarães Nobre et al., 2019; Weingärtner, Pforr, and Wilkinson, 2020). More accurate, local predictive indices for water shortage‐related food insecurity can more robustly justify the ex‐ante (pre‐emergency) financing of anticipatory actions, such as cash transfers, the provision of livestock fodder, short‐term supplemental crop irrigation, and other localised endeavours that mitigate the later costs and impacts of drought (NA1, IA1, IA2, IC2). To exemplify this, two experts (PW1, A1) positively referenced the National Drought Management Authority's early warning bulletins in Kenya, where a deterioration in their local ‘distance to water’ indicator triggers specific water‐focused responses, while also augmenting the holistic indices of drought severity that can initiate broader humanitarian actions (NDMA, 2021).

#### 3.2.2 Market‐based sources of finance

For government and humanitarian sector financing, parametric insurance and other linked securities such as catastrophe bonds have already become important financial risk transferring tools to cover high impact, low probability events (Clarke and Vargas Hill, 2013; Durand et al., 2016; Okpara et al., 2017; Ikeda, 2021). Market‐based finance is also growing in relation to the financing of WRM institutions and water utilities as their creditworthiness for debt finance improves (NW1, NW2). Parametric insurance (or Islamic finance alternatives such as Takaful) for drought has not yet scaled up in the Horn of Africa, particularly for smallholders and district‐level institutions. This is principally owing to the high basis risks faced by both insurers and policyholders, resulting from the current dependence of these products on low‐resolution, low‐confidence satellite‐based indices (NF1, IA1) (Clarke and Vargas Hill, 2013; Durand et al., 2016; Timmons Roberts et al., 2017; Fava and Vrieling, 2021). Four experts (PW1, NF1, ND2, IA1) suggested that in‐situ water security data are required to index more accurately precise parametric drought products, facilitating rapid payouts from pre‐positioned pools of contingency finance once selected indices exceed locally‐established thresholds. By reducing uncertainty and the basis risk of impact miscalculation, high‐resolution datasets can improve the economic viability of providing insurance and contingency credit to water authorities, agricultural asset holders, smallholders, households, business owners, governments, and humanitarian organisations (ND2, NF1, IA1).

## 4 DISCUSSION

### 4.1 An integrated role for water security data

The results of this study suggest that water security data, along with analysis of its causal relations with broader humanitarian impacts, can support the planning and early financing of drought impact‐mitigating actions at greater scale by providing a more confident, predictive evidence base for their design and value proposition. We present the findings in an interpretive conceptual framework, visualising a potential flow of water security data in fulfilling both water resource managers' and financing decision‐makers' information needs for their respective activities in managing droughts (see Figure 5). In our framework, water resource managers use water availability, access, and use indices to support more informed planning of complex WRM and humanitarian solutions, such as borehole rehabilitation, cash transfers, and the provision of surge water supply to areas of insecurity. The water security data are then processed according to the value and financing criteria of different institutions to furnish them with a cost–benefit basis for financing those projects ex‐ante.

### 4.2 Changing the nature of drought finance

Ex‐ante, predictive mechanisms of drought‐related disaster risk finance may have advantages over paradigm financing processes, which we conceptually visualise in Figure 6. Through the earlier financing of anticipatory actions with demonstrably positive returns on investment, water security index‐based financing can reduce both the overall humanitarian impact and the cost of managing a drought (Cabot Venton et al., 2012; Hallegatte, 2012;. Clarke and Vargas Hill, 2013; UNICEF, 2015; Costella et al., 2017; Cabot Venton, 2018; Guimarães Nobre et al., 2019; Pichon, 2019; Weingärtner and Wilkinson, 2019; Weingärtner, Pforr, and Wilkinson, 2020; Chaves‐Gonzalez et al., 2022). Index‐based finance can also pay out with significantly shortened lag times when mechanisms are set up to trigger predesigned actions from pre‐positioned financing (Durand et al., 2016; UN, 2016; Timmons Roberts et al., 2017). The use of local data further reduces time lags, by improving the spatiotemporal precision and reducing the scale of financing requests. Smaller financing requests can be met more rapidly, and their targeted nature can enhance their cost‐efficiency.

Motivating financing stakeholders to develop water monitoring systems and associated pre‐positioned mechanisms of finance requires evaluative case studies of projects triggered by water security data (Clarke and Dercon, 2016; UN, 2016; Weingärtner, Pforr, and Wilkinson, 2020). This exemplifies the value of water security‐indexed anticipatory actions to traditional financing stakeholders, while demonstrating the improving economic viability of providing drought‐associated insurance products to market‐based financers (Pandeya et al., 2016). Regularly evaluating anticipatory actions is also critical to ensuring that implementing actors receive due recognition for anticipatory investments. This is especially true given that the benefits of mitigation projects are not as visibly apparent to the public, colleagues, donors, or shareholders as emergency response projects (Depoorter, 2006; Wilkinson, 2012; Wilkinson, Pforr, and Weingärtner, 2020). One participant (UC2) commented:

*To promote more anticipatory action, quantify the impact. If we could say that our current style of response is 10 times costlier than the prevention, this number is quite powerful … and then also, we need to look at how we could showcase good examples and quantify the impact after the project is operated to encourage more anticipatory action in the future*.

The evaluation of anticipatory and index‐based projects is also critical to identify and correct cases where they have not worked, or where they may have generated disbenefits for the organisations or communities involved (Chaves‐Gonzalez et al., 2022; Scoones, Shariff Mohamed, and Taye, 2024). Anticipatory projects have risks similar to other forms of development finance, such as potential aid dependency and erosion of traditional coping strategies, omission of marginalised groups from benefits, miscalculation of payouts in areas of high uncertainty, data gaps in hard‐to‐reach and vulnerable areas, or possible local currency risks if cash or emergency supplies are distributed in ‘false alarm’ scenarios (Guimarães Nobre et al., 2019; Weingärtner and Wilkinson, 2019; Weingärtner, Pforr, and Wilkinson, 2020; Chaves‐Gonzalez et al., 2022). They may also be inappropriate for the local context (Scoones, Shariff Mohamed, and Taye, 2024), for example, commercial insurance and interest‐based debt financing can contravene Islamic finance laws and traditions unless they are adjusted to compliant mechanisms. Gradual, iterative, and community‐centred approaches are necessary to identify and address risks in project designs, with pre‐implementation testing and continued evaluation of indices and their triggers to ensure they are accurate (Costella et al., 2017; Guimarães Nobre et al., 2019). Evaluations should be shared in a public forum such as that of the Anticipation Hub, which has been successful in facilitating lessons‐sharing between innovative projects in the field (Chaves‐Gonzalez et al., 2022; Anticipation Hub, 2025).

### 4.3 Implementing water security monitoring

Creating high‐resolution, regularly updated databases of water security data requires substantial and long‐term investments in data collection. Given the data's potential value to the wider humanitarian and development sectors, WRM institutions need financial support from potential public, private, and non‐profit data users in these sectors to set up and maintain local water monitoring networks. One participant (IW2) underscored:

*It is a difficult process, none of us [water resource managers] currently have enough capacity to make this very large*.

Maintaining local water monitoring systems is technically challenging. While the monitoring of water access and use requires simple survey methods, like those regularly performed by governments and humanitarian organisations for food security (Young, 2021), the monitoring of water availability remains a significant challenge. Groundwater monitoring, for instance, remains scarce following a history of underinvestment, implementation difficulties, and weak WRM capacities for consistent data collection (Veness and Buytaert, 2025). Yet, the feasibility of hydrological monitoring is ever‐improving through advancements in automatic sensor technologies, automatic data transmission, WRM capacities, and innovative arrangements such as citizen science (Buytaert et al., 2016; Ingram and Memon, 2019; Calderwood et al., 2020; Veness et al., 2022). The upscaling of local hydrological monitoring networks has become technically viable where there is sufficient investment, capacity, and incentive among stakeholders (Walker, Smigaj, and Tani, 2021; Nath and Kirschke, 2023).

Institutional embedment of dedicated information management centres is necessary to ensure that long‐term monitoring, analysis, and communication of water security data are maintained. In Somalia, for example, the UN's SWALIM unit is well‐positioned with prior government integration to fulfil this role, but it requires additional technical and staffing capacity to manage a complex, technical monitoring network (SWALIM, 2025). WRM institutions additionally require investments in their staffing, knowledge, and resource capacities to take actions effectively, at scale, upon the release of finance for water‐securing interventions (Alaerts, 2019; Machete and Marques, 2021; UNESCO World Water Assessment Programme, 2022; Ovink et al., 2023). Monitoring institutions will generally have WRM expertise, so they should attempt to integrate it into existing humanitarian information systems such as FSNAU, FEWS NET, or academic research groups, where analytical capacity and incentive should exist to support the analysis of relationships between water security and food security. While causal links between the two were perceived and described by this study's respondents, their relationships and degrees of causality are highly context‐variable and will require the development of new statistical methods to forecast probabilistic food security outcomes from databases of local water security data (Anderson et al., 2021; Young, 2021).

## 5 CONCLUSION

Addressing the role of drought as a primary driver of food security crises in the Horn of Africa requires management approaches that accord greater priority to the mitigation of water insecurity and its cascading secondary impacts. However, our respondents broadly believed that the underfinancing of pre‐ and early‐drought interventions, particularly those focused on water security, has been a persistent barrier to shifting from reactive to proactive management. This delay in financing is linked to a perceived overdependency of existing humanitarian and disaster risk financing mechanisms on food security information ahead of indicators of water security, as there is often a multiple‐month time lag between the onset of hydrological drought and the triggering of finance by food security indicators.

Local water security data collection remains scarce in the Horn of Africa, but decision‐makers identify such information as critical to the planning and financing of projects that mitigate water shortages and their secondary impacts. For project planning, information on water availability, access, and use enables WRM stakeholders to anticipate shortages and take mitigating actions if they have sufficient capacity. Water security data further support an evidence‐based, long‐term design of more drought‐resilient water supply systems.

Regarding the financing of WRM projects, there is a demand for local water security data to support the development of more confident cost–benefit value propositions in submissions for ex‐ante (pre‐emergency) finance. By analysing water security data to define its correlations and causal relationships with other humanitarian indicators, water security indices can serve as predictors of food insecurity, WASH issues, and other secondary impacts of drought. These secondary impacts can then be forecasted under projected scenarios, and the full expected benefits of financing projects that mitigate water insecurity can be more confidently proposed. Given the current evolution of the international early warning and monitoring landscape (Early Warnings for All, 2025; Locks et al., 2025), there is a real‐time opportunity to develop new systems that promote water security data and its potential in humanitarian forecasting.

As for humanitarian financing during drought events, high‐resolution availability of predictive local water security data can shift financing towards faster, index‐based mechanisms. Within those mechanisms, preplanned drought actions tailored to local contexts can receive timely finance once water security indices exceed predefined thresholds. By lowering the uncertainty and risk associated with investments, local data may also attract greater finance from emerging market‐based sources, such as parametric insurance providers, commercial lenders, and private infrastructure investors.

To incentivise financing stakeholders to develop ex‐ante mechanisms, more case studies are required that demonstrate the value of both anticipatory actions and collecting local water security data at high resolution to index them (Veness and Buytaert, 2025). If implemented projects can demonstrate positive cost–benefit analyses, returns on investments, low risk, and positive political value to the implementors and financers, they will encourage further investments in local water security data collection and associated management systems (Depoorter, 2006; Wilkinson, Pforr, and Weingärtner, 2020). Evaluations should equally be shared in an honest and open forum in cases where projects experience challenges or failures, as this can improve the designs, practices, and outcomes of future implementations in an emerging field. If institutional capacities can also be supported in the implementation of projects, a shift towards ex‐ante financing and management of drought risk is feasible, with the potential to mitigate substantially the humanitarian impacts of the drought hazard.

## ETHICS STATEMENT

This paper reports analysis of primary data. The ethics of data collection and analysis were approved by the Science, Engineering and Technology Research Ethics Committee at Imperial College London.

## FUNDING

This research was funded by the Natural Environment Research Council in the UK (NE/S007415/1) and project partners Concern Worldwide (UK).

## Supporting information


**Data S1** Supporting Information.

## Data Availability

The data that support the findings of this study are available on request from the corresponding author. The data are not publicly available due to privacy or ethical restrictions.

## Endnote

^1^
All supplementary materials can be found online in the Supporting Information section at the end of the article.
