# Supplementary material for: Humanitarian management of drought needs better water security data
Source: Disasters. 2025 May 13;49(3):e12687. doi: 10.1111/disa.12687 (PMC12075996; doi:10.1111/disa.12687)
Supplement: Supplementary file 1 — Data S1 Supporting Information. [file DISA-49-e12687-s001.docx]

# Humanitarian Management of Drought Needs Better Water Security Data - Supporting Information

**Contents**

S1 FSNAU Indicators and Alarm Thresholds

S2 Interview Stakeholder Group Descriptions

S3 Extended Analysis of Drought Management Barriers

S4 Bibliography

**S1 FSNAU Indicators and Alarm Thresholds**


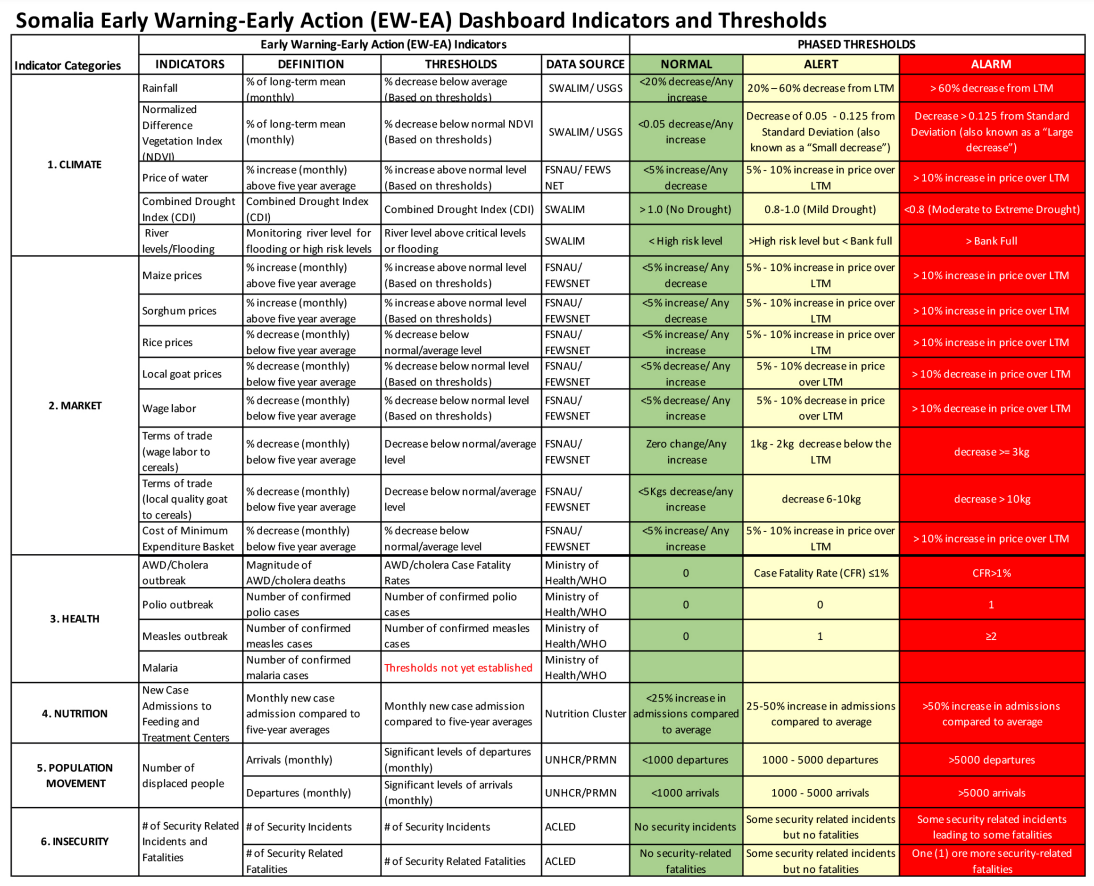


**Figure S1.** The table (from FSNAU, 2023) summarises the indicators displayed on the Food Security Nutrition and Analysis Unit’s Early Warning-Early Action (EW-EA) Dashboard and the threshold criteria for those indicators to be categorised as being in ‘normal’, ‘alert’ or ‘alarm’ phases.

**S2 Interview Stakeholder Group Descriptions**

Interview stakeholder group descriptions are derived directly from the corresponding author’s internally published PhD thesis (Veness et al., 2024).

*S2.1 NGO & IGO*

Non-Governmental Organisation (NGO), Inter-Governmental Organisation (IGO) and United Nations (UN) Water Agriculture Sanitation and Hygiene (WASH) teams support national water authorities in achieving WASH development objectives, often bringing technical expertise and financial capacity that are missing in the institutions that they support. Their objectives align with SDG 6 – clean water access for all. They have a more prominent role in areas of conflict or internally displaced person (IDP) camps to ensure that water is provided to those that cannot afford it.

Analytics teams create value out of physical and socio-economic humanitarian data by turning raw data into outputs and insights that can guide humanitarian, public, private and government decision making. These teams are growing, with prominent examples of FSNAU, FEWS NET, REACH and the Humanitarian Data Exchange (HdX), but their outputs have been criticised for poor communication and failure to tailor to stakeholder needs (Maxwell et al., 2021).

Development, coordination and emergency respondents have wider objectives of meeting all SDGs more broadly. For development workers this ranges from implementing long-term projects such as female-led microenterprises through to investment in more resilient WASH systems – projects are designed to local needs. Emergency responders, in contrast, will be deployed on short-term bases when lifestyles are disrupted following crises such as floods, droughts, locust outbreaks and civil conflicts.

Finance teams control budgets from the international to individual district scale. At all scales, their objective is to fund projects by priority of development or humanitarian needs. As a result, these teams are interested in data sources that can improve the accuracy of decision making to ensure the correct projects are funded to meet local objectives and need.

*S2.2 Water authorities and Government*

National water authorities, often termed ministries, vary in technical, financial and staffing capacity between states, though all of them are the formal lead body in coordinating national water resource management. As a public body they report to politicians and the public, so follow the primary objective of optimising water resource management for the public good. In contrast to NGO and UN WASH professionals, the national authority can enforce government legislation on the public and the private sector when required.

The district water authority and governance of water resources varies between geographies. In states with stronger governance, this may be a district office of the national water authority, a dedicated river basin authority or an urban water utility. In areas of weaker state-governance, this may be a self-organised private or community-owned network. Whether for profit or public service, these institutions’ primary interest is the maintenance of reliable supply, and they have extensive local knowledge, experience and contacts. In the Horn of Africa, these tend to be district arms of the national water authority, and these were interviewed in Somaliland, Kenya, Uganda and Ethiopia in this study. Whilst community-level interviews were not formally conducted for this regional-scale study (though, the lead author did complete fieldwork and informal community member conversations in Somaliland with supporting NGO Concern Worldwide during project ideation), the district water authority representatives spoke frequently about community processes and perspectives from their experiences.

Other government departments, such as departments of planning, development and agriculture are also interested in water resource management, as it affects their ability to meet their own objectives. Representatives from these are frequently drawn into emergency disaster committees during times of drought or other crises to collaboratively design emergency response.

*S2.3 Academia and Private Consultants*

Academic and private consultant experts in WASH and WRM have a combination of core knowledge, expertise and experience that provides rich insight in interview. The academics in this study were both locally and internationally based, while the expert consultants interviewed had a minimum of 15 years’ experience of WASH in SSA.

**S3 Extended Analysis of Drought Management Barriers**

Thematic analysis of expert-perceived barriers to drought management organises them in to 6 themes, each analysed in turn in this section (extracted from the corresponding author’s PhD thesis; Veness, 2024). As an overview, existing monitoring information systems are the modally identified barrier to more effective drought management among experts. At all stakeholder levels, the scarcity of hydrological and regular socio-economic monitoring data is considered a bottleneck to timely, evidence-based decision making. For Water Resource Managers (WRMs), hydrological monitoring scarcity is a barrier to planning evidence-based, ex-ante interventions that maintain water supply. For financing stakeholders, the scarcity of both hydrological and socio-economic monitoring data means that there is not a sufficiently confident evidence-base to justify a forecast-based, ex-ante financing of drought-associated actions. Instead, financing stakeholders wait for socio-economic impacts of water shortages to cause significant increases in indicators hosted by EWSs before releasing drought financing at significant scale. Information scarcity, therefore, also underpins the financing barrier to drought management. The capacity barrier is closely tied to financing, as underfunded Water Resource Management (WRM) institutions are constrained by understaffing, a lack of equipment and low technical capacities. Experts also highlight a range of physical, political and humanitarian access barriers to drought risk management.

*S3.1 Information Systems*

88% of experts raise existing information systems as a barrier to drought management. They are critical of a prevailing monitoring scarcity, the choice of indicators available on platforms, a lack of water security monitoring, and poor communication of existing data.

Leading EWSs in Somalia, including the Famine Early Warning Systems Network (FEWS NET) and the Food Security Nutrition and Analysis Unit (FSNAU), are criticised for spatially and temporally over-aggregating data, creating discrete food security classifications that can over-simplify or incorrectly represent humanitarian needs [NA1, ND3, NE1, PW3, UA1, UA2, UC1, UC2, UW4].

*““oh, we’ve got more red zones than green zones than normal!”. I think that those sorts of early warning systems, exactly the kind that the FSNAU dashboard looks like, are kind of a joke.”*

-IA1

*“I realised that the big systems were not granular enough to tell the difference from this place and that place; they weren't coming in at the right time. I couldn't like dig-in and know whether to really trust that data, or that analysis.”*

-IA2

One expert colloquially describes summary maps produced by these systems as a “curse of the red blobs” [PW1] that have made a humanitarian system “enamoured with food security” [A1], unwilling to fund significant drought-associated actions until real-time food security turns red on district-wide scales on Integrated Phase Classification (IPC) maps [A1, MN3, NA1, NA2, ND1, IA1, IC2].

*“a problem with the current system as it's too broad brush. You know, the indicators will give you an error on a district wide approach, but it won't tell you that this particular community is going to go downhill first or, you know, this is what's going to happen here. And I think that much more localised monitoring, with local triggering of local action is what's really needed.”*

-PW1

The low frequency of data available on these platforms [NA1, ND1, ND3, IA1, IC1, IC2, IW4] and the opacity of the source information behind discrete classifications [ND2, PW3, IA2, IA1, IC2] mean that there is a further lag time until management interventions can be confidently planned and financed. A lack of timely, granular and confident evidence of humanitarian and operational needs is considered a leading factor behind the financing barrier (Section 2.3.1.2) - a lack of evidence for value propositioning drought-mitigating actions bottlenecks their financing [MD4, ND5, NW1, NW2, NW4, PW4, IA1].

*“if we do not have evidence that we can show to donors, how are we going to persuade them to finance special projects to manage the drought?”*

-ND5

An acute scarcity of local water security monitoring data on information platforms is a barrier to WASH professionals, WRMs and drought coordinators when conducting population needs analyses. They consider this data to be undervalued and subsequently underfunded [MD4, NW2, NW4, NW5, PW5, IC1, PW1, A1].

*“we want to organise ourselves at the National Platform and know how to reach those people before they are displaced, because it is actually cheaper to assist people who are lacking water in their villages than to wait until they migrate. Otherwise, their needs are going to increase; maybe they now need shelter, they will need food, they need other things. So, we want to try and actually be proactive in that way.”*

-NW1

The absence of water security data limits WRMs’ abilities to plan appropriate early interventions that maintain water access [A3, MD3, MD4, MN1, ND4, NW4, PW5, IC1]. For example, the absence of groundwater level and quality monitoring means water shortages are rarely anticipated before supply failures occur [MD3, ND2, ND3, NF1, NW1, NW3, PW5, IA1, IC2]. In the long-term, this missing data also constrains the identification of productive aquifers for more sustainable borehole siting [MD2, MD3, MD4, MN3, ND2, NDF2, NW2, NW5, IW2]. As a result, supply failures in poorly designed infrastructure are unmitigated and widespread during droughts, with reactive solutions designed upon reports of failures [GO1, MN3, MD2, A1].

*“at the moment, I still feel everything is reactive.”*

-GO1

*“whenever the droughts come, it’s a repeat cycle - water trucking, water trucking, water trucking.”*

-MN3

*“the emergency approach is to resort to trucking pretty quickly, which is very expensive and inefficient.”*

-A1

Although increasingly available, satellite remote sensing datasets are perceived to be too coarse, inaccurate and uncontextualized to be actionable as confident indicators of local water availability to guide WRM decisions [ND2, ND3, ND5, NW4, PW3, IA2, IC1, IW1, IW2].

*“we have found many times that NDVI [the Normalised Difference Vegetation Index] can be misleading. There are sometimes increases or decreases in places that do not match what we see when we visit the sites.”*

-ND2

*“I am concerned about the way that it is moving – it feels like more and more funding is going in to satellite data analysis.”*

-NW1

Of the WRM-relevant data that is collected, existing EWSs do not tailor communication to WRM stakeholder needs and alternative systems for socio-hydrological information sharing are absent or poorly developed [PW3, PW4, MD4, NA1, ND5, NW4]. Indices such as rainfall anomalies, NDVI and water prices are presented in aggregated, coarse and opaque formats on existing information platforms, with few tools for interpretation or analysis [IA1, PW1, IC1, ND2, NA2, NA1, A1] (Haile, 2005).

Higher-level stakeholders within government, development, humanitarian and donor institutions also feel their information needs are not being met during droughts by existing information systems, and they are frustrated by EWSs’ failures to adapt in response to evaluation and feedback [ND3, PW3, IA1, NW1] (Maxwell et al., 2021).

*“I'm very unhappy with the FSNAU, as it is right now. I think it's stuck. It's not open to the changes that are needed to bring it up to date.”*

-PW3

*“we haven't seen huge jumps forward in how early warning works in Somalia; we are still very much driven by personalities.”*

-IA1

The information system barriers experienced by higher-level financing decision-makers link closely to the financing barrier (MD4, ND5, NW1, NW4, PW4, PW5, IA1].

*S3.2 Financing*

*“a no regrets approach has not been happening this year. Somalia is really struggling with funding - the HRP [Humanitarian Response Plan] is currently funded at 3.8% [of the requested amount].”*

-NF2

Despite broad awareness that earlier actions have vastly improved cost-benefit analyses (CBAs) and returns on investments (ROIs) compared to humanitarian responses, they remain underfunded for droughts [NF2, IC1, ND5, NE2, NF1, NF2, PW1, IC2] (Cha et al., 2018).

*“as a coordinator, often we cannot take anticipatory action, not because we are not anticipating. But because we cannot take action. Because anticipating is not rocket science - if two rains fail, and the next one is failing, you have already 100% anticipated. But, just, we cannot raise the money.”*

-IC1

The paucity of socio-hydrological data and the food security focusses of existing information systems mean that drought financing still depends on perceptible evidence of significant food insecurity for their financing at scale [PW1, IA1, IA2, NA1].

*“there’s still a prevailing perspective that people want to see the red on the map. They want to see the desperate situation on the ground before they will actually take any action. And this is particularly true in terms of government because they have limited funds set aside for emergency response.”*

-PW1

*“I gave a presentation to the team, like a month after I joined, because the Somalia trigger was insane. I mean, it was just based off, I think, IPC [Integrated Phase Classification of food security] warnings, and the Ethiopia trigger was basically just based off IPC classifications. That is like, not just acting after meteorological drought, but it’s acting even after the impact – for early action!*

-IA2

The financing of proactive, ex-ante actions focussed on securing water supply are particularly obstructed by the scarcity of water security data [PW3, PW4, PW5, IC1, IC2]. Without these data, cost-benefit analyses (CBAs) of actions cannot be confidently formulated for financing requests [IC2, NF1, NF2, A1, A2, NA1, NA2, ND2, ND3, NE1, NE2, NW1, PW1]. Current and forecasted water security cannot be calculated with confidence, and the extent to which water security will impact food security and broader humanitarian outcomes cannot be locally defined without historic water security data to identify correlations and causalities [NA1, NA2, PW1, IA1, A1, A2] (Wilhite et al., 2007). Therefore, the full benefits of taking water shortage-mitigating actions cannot be quantified for a CBA [PW4, IC2]. Without confident CBAs of projects to attract finance from limited capital pools (Basolo et al., 2009), finance is instead channelled to other regions, countries or humanitarian crises until measurable humanitarian need has sufficiently increased in the drought-affected region [A1, NE2, NF2, IC2, IA1].

*“the humanitarian sector in general doesn't like the idea of uncertainty. They need something to be concrete, yet Early Warning Early Action is, inherently, embracing uncertainty in some ways, right?”*

-NA1

*“I was previously doing a lot of the nitty gritty kind of analysis for early warning. I had a pot of money, and I was trying to decide, do I use my pot of money now? Or do I hold that pot of money for a more rainy day, so to speak. This put me in a nitty gritty war with FSNAU, essentially trying to triangulate if the things they were saying were right in my areas.”*

-IA1

The absence of regular water security data collection explains the scarcity of dedicated, pre-positioned financing mechanisms for drought management [ND5, ND1, ND2, NE1, NF1, IC2]. Ex-ante financing systems are index-based, requiring accurate indices with low uncertainties to trigger pay-outs once their values exceed pre-set thresholds (Durand et al., 2016). However, when indices have a high uncertainty, there is a high basis risk for both the financing and receiving parties, where pay-outs can be misrepresentative of needs [NF1, NA1, ND2, ND3, NE2, IA1, IA2]. This high basis risk currently constrains interest in existing parametric insurance and insurance-linked securities for drought, as they are indexed by low-resolution, high-uncertainty satellite-based indices [NF1] (Clarke and Hill, 2013; Durand et al., 2016; Ikeda et al., 2021; Okpara et al., 2017).

The transition of drought finance to ex-ante mechanisms is also inhibited by a global scarcity of evaluated case-studies, where the value of ex-ante actions has been quantified in drought areas, to motivate the routine financing of these actions based on established general principles [IC2, PW3, PW4] (Dasgupta, 2021). The scarcity of high-resolution water security data is responsible for the paucity of evaluated examples, as it is not possible to fully evaluate the impacts of ex-ante drought interventions without these data [IC2, PW3, PW4].

*“donors do not give us give us a lot of funding, right? Because anticipatory action, it's very difficult to prove the result. It's not really visible without the right data. But if we have, say, people dying, and we do massive water trucking, it's really catchy, right? Media-wise.”*

-IC2

Funding lag-times in early drought phases allow unmitigated water shortages, triggering water scarcity, food shortages [A1, NA1, ND2, ND3, ND5, IA1, NF1] and displacements [IA1, PW1, NW1, A1, MN3, ND3, NF1, NW2, PW2]. Humanitarian needs, management costs and resource scarcity also compound following late financing [IW3, NE1, PW3, PW4, PW5, IA1, NA1]. For example, the failure of boreholes increases the costs of supplying emergency water from the functional point sources that remain, risking shortages at strategic sites and internally displaced person (IDP) facilities through the ensuing stages of drought [IW3, NE1].

*“the water prices for trucking in Somaliland are now up, in some areas, to 20 – 30 $US/m3. And again, even if we compare it with let's say the UK, the price is still about 2 $US/m3. In Nairobi, around 1 $US/m3.*

-IW3

*“we have to stock water trucking for 45,000 people in the next couple of days, but as of now I still do not have any source [of money] to continue this very crucial activity. So, we may have to exit prematurely, and that will be serious, you know, particularly for the displaced population.”*

-NE1

*S3.3 Capacity*

Low technical, staffing and resource capacities of WRM institutions are a barrier to maintaining drought-resilient water networks. These institutions are underfunded, especially relative to humanitarian programmes, yet they are principally responsible for managing and maintaining clean water supply.

*“it's the capacity of organisations to actually use information and be adaptive, etc. that is actually holding this thing back.”*

-IA1

A shortage of resources, staff and equipment prevent WRMs from taking some desired water-securing actions [MD1, MD4, ND4, ND5, NE1, NW4, UW3, A1, A3, MN2, ND2, NW2, PW3, PW4, IC2, IW2]. Their capacity to design complex water supply solutions is also inhibited by low technical skills, as sustainable, resilient solutions for complex supply systems require a commitment to water monitoring, diagnostic testing, analysis and evidence-based design [A3, GO1, MD1, MD2, MD3, MD4, MD5, MN2, NA1, NA2, ND4, NW1, NW4, PW1, PW3, PW4, IA1, IC2, IW2].

Low capacity limits the reach of WRM institutions, enabling poor water practices to become commonplace among private, community and district engineers [PW3, PW4] (MacAllister et al., 2022; Upton et al., 2017). Without a water authority routinely monitoring or maintaining a significant local presence, unregulated water infrastructure development creates low resilience supply networks that fail quickly under drought conditions when demand increases [MD4, MD5, MN3, ND1, ND4, ND5, NE1, NW1, NW2, PW3, PW4, IW2] (Foster et al., 2019; Kebede et al., 2022).

*“these institutions don’t have the capacity to regulate or penalise contractors who cut corners.”*

-PW4

*S3.4 Physical*

Physical shortages of water are considered a characteristic of the drought hazard rather than a major barrier to its management. In deep boreholes, water supply rarely fails due to the depletion of water availability, but frequently, these boreholes fail due to the transfer of demand from shallow and surface sources onto strained infrastructure [MD1, PW3, PW4, MD4, PW5, A1] (Calow et al., 2010). However, in remote areas with poor aquifers, such as southern and eastern Somaliland, genuine water shortages in accessible, freshwater aquifers are a barrier to siting boreholes with an adequate water availability [MD5, MN3, ND5, ND1, IW3].

*S3.5 Humanitarian Access*

The most vulnerable communities to drought are often in conflict areas, remote settlements, or those actively migrating. When water and food supplies fail in remote or conflict areas, humanitarian assistance may not be accessible [MD4, ND5, NE1, NF1].

*“with migration comes mortality, especially for children.”*

-NE2

Experts identify the shortage of real-time migration data, and the current difficulty in predicting migration source areas and routes, as a barrier to supporting the vulnerable and displaced [MD4, MN3, ND3, NE1, NE2, NF1, NW1, NW2, PW2, IW1]. They cite a lack local food and water security data to anticipate migration source areas and routes in advance [NE1, NW2, IW1]. Migration that exceeds clan or tribal boundaries needs more anticipatory mitigation, as it is seen as a cause of flash-escalation in vulnerability and humanitarian needs when migrating communities are subjected to marginalisation and conflict [IA1, A1].

*“you don't want your anchor water points to fail. Because when your anchor points fail, you get people migrating outside of their normal clan lands and areas. And that tends to be when really bad things happen.”*

-IA1

*S3.6 Political*

Conflict is equally viewed as a political barrier, compromising public services, trade and humanitarian activities that support the drought-affected [NE1, ND5]. Political bureaucracy surrounding early warning interpretation, and when to officially declare drought or famine, is also a short-term bottleneck on the release of funding by governments and humanitarian organisations [GO1, NF2, IA2, MW2, NE2, PW3, IC2] (Maxwell et al., 2023). There can be hesitancy among governments to formally declare emergencies, especially in election years, as it may compromise their public image [NF2, IA2]. This can lead to costly funding delays while humanitarian needs escalate.

*“some of the grants and resources come available after declaration of drought, and this is kind of common in many other countries also, not only in Somaliland, for example, when the government declares that there is a drought emergency, then some of the international community will start to allocate some funds and resources.”*

-IW3

Public image considerations inhibit the financing of longer-term, resilience building or anticipatory solutions even when a positive return on investment is clear [IW3, NA1, IC2] (Wilkinson et al., 2020). Political myopia creates preference for short-term benefits [IW3]. There is also a reluctance to dedicate resources to disaster mitigating actions if it does not earn recognition among voters or colleagues [IC2, IW1, IW3, NA1] (Depoorter, 2006).

**S4 Bibliography**

Basolo, V., Steinberg, L. J., Burby, R. J., Levine, J., Cruz, A., & Huang, C. (2009). The effects of confidence in government and information on perceived and actual preparedness for disasters. *Environment and Behavior.* 41(3), 338-364.

Calow, R. C., MacDonald, A. M., Nicol, A. L., & Robins, N. S. (2010). Ground Water Security and Drought in Africa: Linking Availability, Access, and Demand. Groundwater, 48(2), 246–256. <https://doi.org/10.1111/j.1745-6584.2009.00558.x>

Clarke, D. & Hill, R. V. (2013). *Cost–benefit analysis of the African Risk Capacity Facility, IFPRI Discussion Paper 01292*.IFPRI. <http://ebrary.ifpri.org/cdm/ref/collection/p15738coll2/id/127813>

Dasgupta, P. (2021). *The Economics of Biodiversity: The Dasgupta Review.* HM Treasury. <https://assets.publishing.service.gov.uk/government/uploads/system/uploads/attachment_data/file/962785/The_Economics_of_Biodiversity_The_Dasgupta_Review_Full_Report.pdf>

Depoorter, B. (2006). Horizontal political externalities: the supply and demand of disaster management. *Duke Law Journal* 56: 101–125.

Durand, A., Hoffmeister, V., Weikmans, R., Gewirtzman, J., Natson, S., Huq, S. & Roberts, J. T. (2016). *Financing Options for Loss and Damage : a Review and Roadmap. Discussion Paper.* German Development Institute. <https://www.idos-research.de/uploads/media/DP_21.2016.pdf>

Foster, T., Furey, S., Banks, B. & Willetts, J. (2019). Functionality of handpump water supplies: a review of data from Sub-Saharan Africa and the Asia-Pacific Region. Int. J. Water Resour. Dev., 1 – 15. <https://doi.org/10.1080/07900627.2018.1543117>

FSNAU. (2023). *Somalia Early Warning – Early Action (EW-EA) Dashboard Indicators and Thresholds.* <https://dashboard.fsnau.org/application/cache/images/EWEA_Dashboard_Indictor_Thresholds.pdf>

Haile, M. (2005). Weather patterns, food security and humanitarian response in sub-Saharan Africa. Philosophical Transactions of the Royal Society B: Biological Sciences, 360(1463), 2169–2182. https://doi.org/10.1098/rstb.2005.1746

Ikeda, J. (2021). *Guidance note on Drought Finance. Innovative Financial Instruments for Drought Mitigation, Preparedness, Response and Recovery.* <https://www.unccd.int/sites/default/files/2022-09/IWG%20task%20group%20report%203%20Drought%20finance%20.pdf>

IMF. (2011). *International Monetary Fund, Market-Based Instruments for International Aviation and Shipping of Climate Finance.* <https://www.imf.org/external/np/g20/pdf/110411a.pdf>.

Law, J. (1987). Technology and Heterogeneous Engineering: The Case of Portuguese Expansion. MIT Press.

Law, J. (1992). Notes on the theory of the actor-network: Ordering, strategy, and heterogeneity. Systems Practice, 5(4), 379–393. <https://doi.org/10.1007/BF01059830>

MacAllister, D. J., Nedaw, D., Kebede, S., Mkandawire, T., Makuluni, P., Shaba, C., Okullo, J., Owor, M., Carter, R., Chilton, J., Casey, V., Fallas, H., & MacDonald, A. M. (2022). Contribution of physical factors to handpump borehole functionality in Africa. Science of The Total Environment, 851, 158343. <https://doi.org/10.1016/j.scitotenv.2022.158343>

Maxwell, D., Day, M. & Hailey, P. (2023). *Do Famine Declarations Really Lead to Increased Funding?* Tufts University. <https://fic.tufts.edu/publication-item/do-famine-declarations-really-lead-to-increased-funding/>

Maxwell, D., Lentz, E., Simmons, C., & Gottlieb, G. (2021). *Early Warning and Early Action for Increased Resilience of Livelihoods in IGAD Region*. Tufts University.

Okpara, J. N., Afiesimama, E. A., Anuforom, A. C., Owino, A., & Ogunjobi, K. O. (2017). The applicability of Standardized Precipitation Index: Drought characterization for early warning system and weather index insurance in West Africa. Natural Hazards, 89(2), 555–583. <https://doi.org/10.1007/s11069-017-2980-6>

Upton, K., Healy, A., Allan, S., Bristow, G., Bukar, Y., Capstick, S., Danert, K., Goni, A., MacDonald, M., Tijana, S., Theis, L. & Whitmarsh, L. (2017) *Risks and resilience of private boreholes in Lagos, Nigeria.* [*https://nora.nerc.ac.uk/id/eprint/519948/1/RIGSS_InesonLecture_2017.pdf*](https://nora.nerc.ac.uk/id/eprint/519948/1/RIGSS_InesonLecture_2017.pdf)

Veness, W. A. (2024). *Towards Resolving Data Scarcity in Water Resources Management* [Doctoral dissertation, Imperial College London]. Spiral Digital Repository.

Wilhite, D. A., Svoboda, M. D., & Hayes, M. J. (2007). Understanding the complex impacts of drought: A key to enhancing drought mitigation and preparedness. Water Resources Management, 21(5), 763–774. <https://doi.org/10.1007/s11269-006-9076-5>

Wilkinson, E., Pforr, T., & Weingartner, L. (2020). *Integrating ‘anticipatory action’ in disaster risk management.* <https://cdn.odi.org/media/documents/202004_odi_anticipatory_action_bn_revised.pdf>
